# Factors Associated with the Technical Success of Bilateral Endoscopic Metallic Stenting with Partial Stent-In-Stent Placement in Patients with Malignant Hilar Biliary Obstruction

**DOI:** 10.1155/2019/5928040

**Published:** 2019-09-16

**Authors:** Toshihiro Fujita, Shinichi Hashimoto, Shiroh Tanoue, Kengo Tsuneyoshi, Yoshitaka Nakamura, Makoto Hinokuchi, Hiromichi Iwaya, Shiho Arima, Yuji Iwashita, Fumisato Sasaki, Hiroki Taguchi, Shuji Kanmura, Akio Ido

**Affiliations:** Digestive and Lifestyle Diseases, Kagoshima University Graduate School of Medical and Dental Sciences, 8-35-1 Sakuragaoka, Kagoshima 890-8520, Japan

## Abstract

**Background:**

Bilateral biliary drainage decreases the risk of cholangitis, but bilateral endoscopic metallic stenting is technically challenging.

**Aim:**

We retrospectively evaluated the factors associated with successful bilateral self-expanding metal stent (SEMS) placement using the partial stent-in-stent (PSIS) method for malignant hilar biliary obstruction and also assessed the safety and efficacy of this technique.

**Methods:**

From April 2010 to February 2016, 47 consecutive patients (mean age, 73.0 ± 8.6 years; 32 males and 15 females) underwent PSIS placement for malignant hilar biliary obstruction in our hospital. The technical success of PSIS, clinical response, and complications were investigated. Factors associated with the technical success of PSIS were assessed. Using a propensity score-matched analysis, we compared the procedure time, clinical response, complications, stent patency, and survival time in 17 matched patients treated with bilateral SEMS placement using a SEMS delivery system of <6.0 or ≥6.0 Fr.

**Results:**

The technical success rate was 77%. The clinical response rate was 91%, and the complication rate was 26%. Regarding complications, pancreatitis occurred in 5 patients (11%), cholangitis in 6 (13%), and cholecystitis in 1 (2%). A multiple logistic regression analysis identified the use of a SEMS with a delivery system < 6.0 Fr as a factor associated with technical success (*P* = 0.033; odds ratio, 10.769; 95% confidence interval, 1.205-96.212). In the 17 matched patients assigned according to the SEMS delivery system size, the procedure time was significantly shorter in those with a delivery system size < 6.0 Fr than in those with ≥6.0 Fr (*P* < 0.01). There were no significant differences in the clinical response, complication rate, stent patency, or survival time between the two groups.

**Conclusion:**

Using a delivery system < 6.0 Fr in size helped improve the technical success and reduced the procedure time for the placement of a SEMS by the PSIS method.

## 1. Introduction

Whether unilateral or bilateral self-expandable metallic stent (SEMS) placement is preferable in patients with malignant hilar biliary obstruction (MHBO) is controversial. Many endoscopists have attempted bilateral SEMS placement because bilateral SEMS placement is more physiological than unilateral placement, thereby preventing obstructive cholangitis [1–4]. Furthermore, evidence suggests that bilateral endoscopic drainage has a longer stent patency than unilateral drainage [2, 5, 6]. In a recent study, Lee et al. reported that bilateral SEMSs had not only merits with regard to stent patency but also low reintervention rates [7].

Bilateral endoscopic metallic stenting can be achieved by the partial stent-in-stent (PSIS) method and the side-by-side (SBS) method. The stent patency and survival time were similar between the two methods [8]. In Japan, many endoscopists have attempted the PSIS method, showing a technical success rate of 81.8%-100% [8, 9], but they have often encountered difficulty inserting the second SEMS through the mesh of the first stent with this method.

No study has yet evaluated the factors associated with successful bilateral SEMS placement via the PSIS method. We therefore retrospectively evaluated the factors associated with successful bilateral SEMS placement using this method for MHBO and assessed the safety and efficacy of this technique.

## 2. Materials and Methods

### 2.1. Patients

From April 2010 to February 2016, SEMS placement with the PSIS method for MHBO was attempted in 54 consecutive patients in our hospital. Patients were excluded from this study if they had been previously treated with SEMS placement (*n* = 5), received SEMS placement by the SBS method (*n* = 1), or previously received gastric surgery with gastrointestinal reconstruction (*n* = 1). After these exclusions, 47 patients (mean age, 73.0 ± 8.6 years; 32 males and 15 females) were included in the analysis.

Diagnoses of malignancy were histologically confirmed by tissue samples. If tissue samples were not available, then the diagnoses of malignancy were confirmed by the combination of clinical, laboratory, and radiologic findings. In the study, patients were considered to have unresectable tumors if they had radiological findings of massive horizontal tumor extension of the bile duct and/or metastasis, an insufficient predicted remnant liver volume, or a poor general condition.

All patients provided their written informed consent, and this study protocol was approved by the ethics committee of our hospital (approval number 27-195) and conducted in accordance with the Declaration of Helsinki.

### 2.2. Endoscopic Procedures

We usually perform the PSIS method for patients with MHBO. In the present study, endoscopic procedures were performed under conscious sedation with diazepam, midazolam, and pentazocine; the doses of which depended on the patient's age and general condition. Prophylactic treatment with broad-spectrum antibiotics and nafamostat mesilate was initiated after the procedure. A TJF240, JF260V (Olympus Medical Systems, Tokyo, Japan), was used for endoscopic retrograde cholangiopancreatography (ERCP) in this study. After biliary cannulation, the location and length of the stricture were evaluated by cholangiography. Two guidewires (Visiglide2 (Olympus Medical Systems) and/or Jagwire (Boston Scientific Corp., Natick, MA, USA)) were advanced one each into the left hepatic duct and anterior or posterior branch of the right hepatic duct through the stricture. If the guidewires did not pass through the sites of biliary stenoses, then a hydrophilic guidewire (Radifocus (Terumo, Tokyo, Japan) or Navipro (Boston Scientific Corp.)) was used to negotiate through the sites of stenoses. The first stent was inserted and placed over a guidewire via the working channel of the scope. The guidewire utilized for the deployment of the first stent was then inserted into the contralateral side via the open mesh of the first stent based on information regarding the configuration of the second guidewire under fluoroscopic guidance. After successful insertion of the first guidewire into the opposite side, the second guidewire was retrieved. Finally, the second metal stent was deployed over the guidewire into the contralateral hepatic duct. A dilation catheter (7, 8, 10 Fr, Gadelius Medical, Tokyo, Japan) and/or balloon 6 or 8 mm in diameter (Hurricane RX; Boston Scientific) was used for the dilation of both the mesh and inner lumen of the first stent if the first guidewire and second SEMS were difficult to insert into the opposite side via the first stent. Unilateral stent placement was performed if the initially planned PSIS placement failed.

Fourteen endoscopists performed the procedure in this study. The trainee usually started the endoscopic procedure; however, the experts (S. H, S. T, and H. T) replaced the trainee when the trainee was unable to complete the procedure. The kind of SEMS to be placed was left to the discretion of the endoscopist and institution. The SEMSs used in this study were WallStent (*n* = 3, Boston Scientific Japan K.K., Tokyo, Japan), Niti-S large cell D-type (*n* = 1, Century Medical, Tokyo, Japan), X-suit NIR (*n* = 10, Olympus, Tokyo, Japan), Zeo stent (*n* = 10, Zeon Medical, Tokyo, Japan), Bonastent M-hilar (*n* = 1, Medico's Hirata Inc., Tokyo, Japan), Zilver 635 (*n* = 4, Cook Medical Endoscopy, Winston-Salem, NC, USA), and BileRush Selective (*n* = 18, Piolax Medical Devices, Inc., Yokohama, Japan) (Table 1). All SEMSs were uncovered type. In all patients who received SEMS placement with a delivery system < 6.0 Fr, BileRush Selective of 10 mm in the stent diameter was used for the bilateral stenosis of the hilar biliary tree.

### 2.3. Outcome Measurements

The technical success rate for the PSIS method, clinical response rate for the method, and complications were investigated. Factors associated with the technical success of PSIS were assessed. In patients treated with bilateral SEMS placement, the procedure time, clinical response, and complications were compared based on the SEMS delivery system size (<6.0 vs. ≥6.0 Fr).

Technical success was defined as the success of the planned placement of a bilateral SEMS for the biliary stenosis by the PSIS method. A clinical response was defined as a reduction in the serum total bilirubin level to normal (<1.2 mg/dL) or less than half of the pretreatment level for 2 weeks. The total procedure time of ERCP and stenting time, which was defined as the time from cannulation of the initial SEMS to the complete placement of all SEMS, were calculated by referencing the medical record of the procedure. A diagnosis of post-ERCP pancreatitis was defined according to the consensus criteria [10]. Cholangitis was defined as a condition accompanied by abdominal pain and a fever (body temperature > 38°C) with an elevated serum level of hepatobiliary enzymes from 24 h after the procedure.

### 2.4. The Propensity Score-Matched Analysis

The patients who underwent bilateral endoscopic metallic stenting were divided into two groups by the delivery system size ((i) thin delivery system group: diameter of the delivery systems for both stents < 6.0 Fr and (ii) thick delivery system group: diameter of the delivery systems for either stent ≥ 6.0 Fr). These two groups were matched in a 1 : 1 ratio (<6.0 Fr, *n* = 17; ≥6.0 Fr, *n* = 17) by a propensity score-matched analysis with adjusting for 6 covariates (age, sex, cause of MHBO, drainage before SEMS placement, endoscopic sphincterotomy (EST) before SEMS placement, and Bismuth classification) to minimize inherent bias.

### 2.5. Statistical Analyses

Student's *t*-test and the Mann-Whitney *U* test were used for continuous data comparisons. Fisher's exact test and Pearson's *χ*^2^ test were used for comparisons of categorical data, as appropriate. A multivariate analysis was performed as a multiple logistic regression analysis of factors with *P* values < 0.10 according to a univariate analysis. Propensity scores were estimated using logistic regression. The stent patency and survival time were calculated by Kaplan–Meier curves and log-rank test. A *P* value of <0.05 was considered to be significant in all analyses. For the statistical analyses, the SPSS software program (ver. 22; IBM Corp., Armonk, NY, USA) was used.

## 3. Results and Discussion

### 3.1. Results

**Figure 1:**
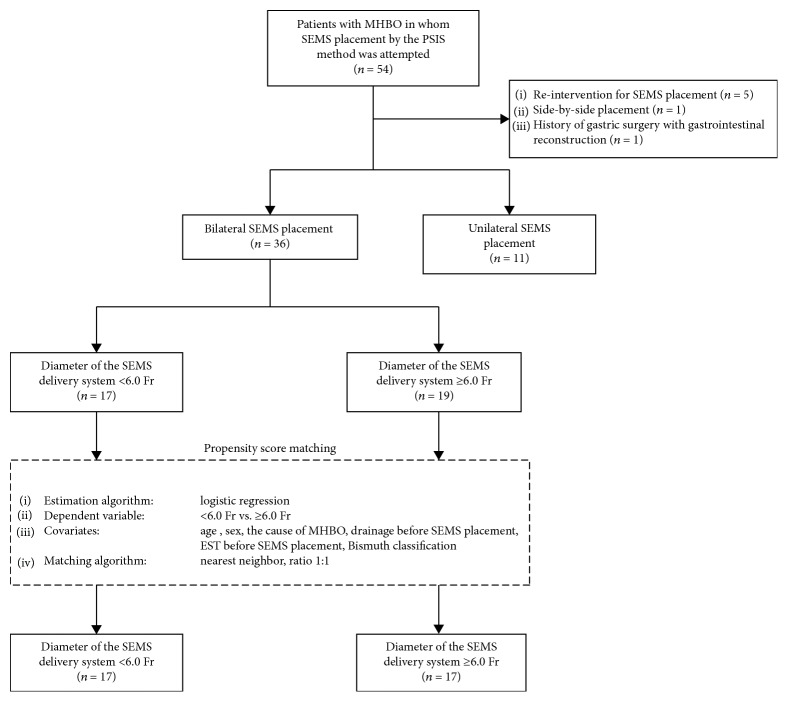
Flowchart of the study population selection and matching by propensity score. MHBO: malignant hilar biliary obstruction; SEMS: self-expandable metallic stent; PSIS: partial stent-in-stent; EST: endoscopic sphincterotomy.

A flow chart of this study is shown in Figure 1. Overall, 47 patients were included in this study. The characteristics of all patients are shown in Table 2. The mean age was 73.0 ± 8.6 years, and there were 32 men (68%). The cause of MHBO was bile duct cancer in 35 (74%), gallbladder cancer in 5 (11%), hepatocellular carcinoma in 4 (9%), metastatic disease in 2 (4%) (primary origin: colorectal cancer and pancreatic cancer), and biliary invasion of gastric cancer in 1 (2%). Drainage before SEMS placement was performed in 34 (72%). EST before SEMS placement was performed in 15 (32%). The Bismuth classification was type I in 0, type II in 12 (26%), type III in 14 (30%), and type IV in 21 (44%). Chemotherapy or radiation was performed before SEMS placement in 10 (21%).

#### 3.1.1. Technical Success and the Factors Affecting the Success of Bilateral SEMS Placement by the PSIS Method

The technical success rate for the PSIS method was 77%. In 11 patients (23%), bilateral SEMS placement was unsuccessful. In nine of these patients, the double guidewires could not pass through the MHBO, and in the remaining two, the second SEMS could not pass through the mesh of the first SEMS.

The clinical response rate for the method was 91%, and the complication rate after stenting was 26%. Regarding complications, pancreatitis occurred in 5 patients (11%), cholangitis occurred in 6 patients (13%), and cholecystitis occurred in 1 patient (2%).

Among the 36 patients who underwent bilateral endoscopic metallic stenting, dilation of the first SEMS was performed in 27 patients (75%). The mesh of the first SEMS was dilated in 9 patients, and the lumen of the first SEMS was dilated in 2 patients. Both of them were dilated in 16 patients. In contrast, all patients who underwent unilateral SEMS placement received dilation of both the mesh and the lumen of the SEMS.

The factors affecting the success of bilateral SEMS placement by the PSIS method are shown in Table 3. There were no significant differences in the patient age, sex, cause of MHBO, existence of biliary drainage before SEMS placement, existence of EST before SEMS placement, Bismuth classification, SEMS type, or use of dilation devices between bilateral and unilateral SEMS placement. In the univariate analysis, the use of a SEMS delivery system < 6.0 Fr in diameter was the only significant factor influencing the technical success (*P* = 0.023). A multivariate analysis confirmed that the use of a SEMS delivery system < 6.0 Fr in diameter was a factor associated with the technical success (*P* = 0.033; odds ratio, 10.769; 95% confidence interval, 1.205-96.212).

#### 3.1.2. Clinical Outcomes of Bilateral Placement of SEMS with the Delivery System Size < 6.0 Fr in Diameter

The patients who underwent bilateral SEMS placement by the PSIS method were divided into two groups based on the delivery system size (thin vs. thick delivery system group). The characteristics of these two groups after propensity score matching are shown in Table 4. The characteristics were similar between the two groups. However, the total ERCP time and stenting time were significantly shorter in the thin delivery system group than in the thick delivery system group (*P* = 0.009 and *P* = 0.009, respectively). There were no significant differences in the rate of using dilation devices (balloon and/or dilation catheter), clinical response, or the complication rate between the two groups (Table 5).

**Figure 2:**
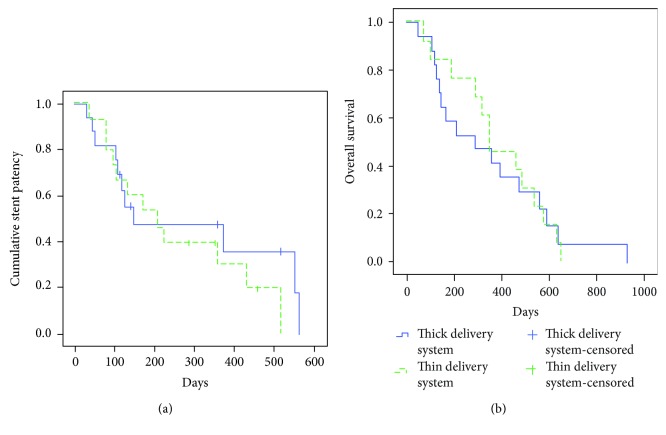
Kaplan–Meier curves revealed no significant difference in the stent patency ((a) *P* = 0.462) and survival time between the 2 groups ((b) *P* = 0.979). The thin delivery system group used a SEMS delivery system of <6.0 Fr, and the thick delivery system group used a SEMS delivery system of ≥6.0 Fr.

The median time of stent patency was 234 and 211 days in the thin and thick delivery system groups, respectively (*P* = 0.462, Figure 2). The 90-day and 6-month stent patency rates were also similar between the 2 groups (93% vs. 82% (*P* = 0.350) and 53% vs. 53% (*P* = 0.982)). Twenty-nine patients (thin delivery system group, *n* = 13; thick delivery system group, *n* = 16) died during the follow-up. The cause of death was tumor progression in 29 patients. Kaplan–Meier curves revealed no significant differences in the stent patency or survival time between the 2 groups (383 days vs. 341 days, *P* = 0.979, Figure 2).

### 3.2. Discussion

We showed here that the use of a SEMS delivery system < 6.0 Fr in diameter contributed to the successful placement of SEMSs using the PSIS method. Bilateral endoscopic metallic stenting is technically challenging. Kogure et al. [11, 12], Lee et al. [13], and Hwang et al. [14] reported high technical success rates with large-cell stents using PSIS deployment. However, failure to insert the second SEMS through the mesh can be caused by insufficient expansion of the first SEMS due to low radial force [15]. Consequently, a large mesh size and strong radial force of the first SEMS are considered factors supporting the success of bilateral SEMS placement by the PSIS method. While many kinds of SEMSs have been used with the PSIS method, which ones are the most suitable has been unclear.

The most difficult part of the PSIS method involves advancing the second stent through the wire mesh of the initial stent. For the introduction of the second stent, the PSIS method for MHBO is often required, not only to negotiate through the site of biliary stenosis but also to dilate the site of biliary stenosis and the mesh of the initial stent [16–18]. Because the thin delivery system could be placed smoothly through the mesh of the initial stent without dilating the wire mesh, this system helped in shortening the procedure time. Kawakubo et al. reported that a laser-cut-type SEMS with a large mesh and thin delivery system was preferable for the PSIS procedure [19]. Our result supported the findings of that study because SEMSs with a thin delivery system (5.7 Fr; BileRush Selective) have a laser-cut-type configuration and large stent mesh. These characteristics of the SEMS contributed to the technical success and shorter procedure time of the present study.

The clinical response, complication rates, and stent patency were similar between the two delivery system groups in this study. Sofuni et al. reported that a procedure time of ≥30 minutes is a risk factor of post-ERCP pancreatitis [20], so reducing the procedure time of ERCP is desired. In addition, there were no significant differences in the stent patency or survival time between the two groups. Given these findings, a thin stent delivery system appears to have merit for patients with MHBO.

Several limitations associated with the present study warrant mention. First, this study was retrospective with an uncontrolled design and was conducted at a single center. The sample size was also quite small. Second, the choice of SEMS was not standardized in this study and was left to each ERCP operator. The cut-off value of 6 or 7 Fr for the diameter of the delivery system for the stent that was used did not significantly affect the success of bilateral SEMS placement by the PSIS method (*P* = 0.138 and *P* = 0.341). The result may be due to the small number cases in which a 6 or 7 Fr delivery system was used. However, the use of a thin delivery system is considered to be effective since it allows the site of biliary stenosis to be easily passed through. In addition, Kim et al. reported that the outcomes were better for techniques performed during the late period than in the early period because the new devices required technical experience [21]. Given that the SEMS with a thin delivery system was not available from April 2010 to January 2015, we cannot exclude the possibility of technical bias with regard to technical immaturity, although early cases of ERCP performed before March 2010 were excluded. A prospective, randomized controlled trial of SEMS placement by the PSIS method for MHBO should be conducted.

## 4. Conclusions

The use of a delivery system < 6.0 Fr in diameter helps improve the technical success and reduce the procedure time of ERCP.

## Figures and Tables

**Table 1:** SEMSs used in this study.

	Diameter of delivery system (Fr)	Type	*n* = 47	
			Diameter of SEMS (8 mm)	Diameter of SEMS (10 mm)
WallFlex Biliary	8	Braided	1	2
Niti-S large cell D-type stent	8	Braided	0	1
X-suit NIR	7.5	Laser-cut	0	10
Zeo stent plus	7.2	Laser-cut	2	8
Bonastent	7	Braided	0	1
Zilver 635	6	Laser-cut	0	4
BileRush Selective	5.7	Laser-cut	0	18

**Table 2:** Clinical characteristics of the 47 patients with MHBO.

	*n* = 47	
Age, mean ± SD (years)	73.0 ± 8.6	
Male/female, *n* (%)	32/15	(68/32)
The cause of MHBO, bile duct cancer/others, *n* (%)	35/12	(74/26)
Drainage before SEMS placement, +/-, *n* (%)	34/13	(72/28)
Existence of EST before SEMS placement, +/-, *n* (%)	15/32	(32/68)
Bismuth classification, II/III, IV, *n* (%)	12/35	(26/74)
Chemotherapy or radiation before SEMS placement, +/-, *n* (%)	10/37	(21/79)

MHBO: malignant hilar biliary obstruction; SEMS: self-expandable metallic stent; EST: endoscopic sphincterotomy.

**Table 3:** The factors for technical success of PSIS.

	Bilateral SEMS placement *n* = 36	Unilateral SEMS placement *n* = 11	Univariate analysis	Multivariate analysis	
			*P* value	*P* value	OR (95% CI)
Age, mean ± SD (years)	73.3 ± 9.0	72.3 ± 8.0	0.741		
Male	26	6	0.229		
Bile duct cancer	28	7	0.285		
Drainage before SEMS placement	10	3	0.647		
EST before SEMS placement	13	2	0.232		
Bismuth classification, type III, IV	28	7	0.285		
Laser-cut-type SEMS	33	9	0.332		
Use of dilation devices	27	11	0.069		
Delivery system size of placed SEMS < 6.0 Fr	17	1	0.023	0.033	10.769 (1.205-96.212)

MHBO: malignant hilar biliary obstruction; SEMS: self-expandable metallic stent; EST: endoscopic sphincterotomy; OR: odds ratio; CI: confidence interval; PSIS: partial stent-in-stent.

**Table 4:** Clinical characteristics of patients with bilateral SEMS placement.

	All participants, *n* = 36			Matched pairs, *n* = 34		
	Used delivery system of SEMS			Used delivery system of SEMS		
	<6.0 Fr *n* = 17	≥6.0 Fr *n* = 19	*P* value	<6.0 Fr *n* = 17	≥6.0 Fr *n* = 17	*P* value
Age, mean ± SD	71.7 ± 8.8	74.7 ± 9.2	0.31	71.7 ± 8.8	74.2 ± 9.6	0.427
Male/female, *n* (%)	13/4 (76/24)	13/6 (68/32)	0.436	13/4 (76/24)	11/6 (65/35)	0.452
The cause of MHBO, bile duct cancer/others, *n* (%)	14/3 (82/18)	14/5 (82/18)	0.414	14/3 (82/18)	12/5 (71/29)	0.344
Drainage before SEMS placement, +/-, *n* (%)	6/11 (35/65)	4/15 (21/79)	0.281	6/11 (35/65)	4/13 (24/76)	0.452
Existence of EST before SEMS placement, +/-, *n* (%)	9/8 (53/47)	4/15 (21/79)	0.047	9/8 (53/47)	4/13 (24/76)	0.151
Bismuth classification, II/III, IV, *n* (%)	5/12 (29/71)	3/16 (16/84)	0.281	5/12 (29/71)	3/14 (18/82)	0.344

MHBO: malignant hilar biliary obstruction; SEMS: self-expandable metallic stent; EST: endoscopic sphincterotomy.

**Table 5:** Outcomes of the two groups.

	All participants, *n* = 36			Matched pairs, *n* = 34		
	Used delivery system of SEMS			Used delivery system of SEMS		
	<6.0 Fr *n* = 17	≥6.0 Fr *n* = 19	*P* value	<6.0 Fr *n* = 17	≥6.0 Fr *n* = 17	*P* value
Procedure time, min (range)						
Total procedure time of ERCP	54.3 (20-132)	81.0 (35-177)	0.004	54.3 (20-132)	86.7 (35-149)	0.009
Stenting time	12.8 (3-23)	22.0 (7-124)	0.003	12.8 (3-23)	26.6 (7-84)	0.009
Use of dilation devices, *n* (%)	14 (82)	13 (68)	0.283	14 (82)	11 (65)	0.219
Clinical response, effective, *n* (%)	15 (88)	19 (100)	0.216	15 (88)	17 (100)	0.242
Complication, *n* (%)	4 (24)	5 (26)	0.577	4 (24)	4 (24)	0.656

SEMS: self-expandable metallic stent; ERCP: endoscopic retrograde cholangiopancreatography.

## Data Availability

The data used to support the findings of this study are included within the article.

## Abbreviations

ERCP: Endoscopic retrograde cholangiopancreatography
EST: Endoscopic sphincterotomy
SEMS: Self-expanding metallic stent
MHBO: Malignant hilar biliary obstruction
PSIS: Partial stent-in-stent
SBS: Side-by-side.

## References

[1] Deviere J., Baize M., de Toeuf J., Cremer M. (1988). Long-term follow-up of patients with hilar malignant stricture treated by endoscopic internal biliary drainage. *Gastrointestinal Endoscopy*.

[2] Chang W. H., Kortan P., Haber G. B. (1998). Outcome in patients with bifurcation tumors who undergo unilateral versus bilateral hepatic duct drainage. *Gastrointestinal Endoscopy*.

[3] Andersen J. R., Sorensen S. M., Kruse A., Rokkjaer M., Matzen P. (1989). Randomised trial of endoscopic endoprosthesis versus operative bypass in malignant obstructive jaundice. *Gut*.

[4] Eisen G. M., Dominitz J. A., Faigel D. O. (2001). An annotated algorithmic approach to malignant biliary obstruction. *Gastrointestinal Endoscopy*.

[5] Liberato M. J. A., Canena J. M. T. (2012). Endoscopic stenting for hilar cholangiocarcinoma: efficacy of unilateral and bilateral placement of plastic and metal stents in a retrospective review of 480 patients. *BMC Gastroenterology*.

[6] Naitoh I., Ohara H., Nakazawa T. (2009). Unilateral versus bilateral endoscopic metal stenting for malignant hilar biliary obstruction. *Journal of Gastroenterology and Hepatology*.

[7] Lee T. H., Kim T. H., Moon J. H. (2017). Bilateral versus unilateral placement of metal stents for inoperable high-grade malignant hilar biliary strictures: a multicenter, prospective, randomized study (with video). *Gastrointestinal Endoscopy*.

[8] Kim K. M., Lee K. H., Chung Y. H. (2012). A comparison of bilateral stenting methods for malignant hilar biliary obstruction. *Hepato-Gastroenterology*.

[9] Kanno Y., Ito K., Fujita N. (2011). Single-session endoscopic bilateral y-configured placement of metal stents for hilar malignant biliary obstruction. *Digestive Endoscopy*.

[10] Cotton P. B., Lehman G., Vennes J. (1991). Endoscopic sphincterotomy complications and their management: an attempt at consensus. *Gastrointestinal Endoscopy*.

[11] Kogure H., Isayama H., Nakai Y. (2011). Newly designed large cell Niti-S stent for malignant hilar biliary obstruction: a pilot study. *Surgical Endoscopy*.

[12] Kogure H., Isayama H., Nakai Y. (2014). High single-session success rate of endoscopic bilateral stent-in-stent placement with modified large cell Niti-S stents for malignant hilar biliary obstruction. *Digestive Endoscopy*.

[13] Lee J. H., Kang D. H., Kim J. Y. (2007). Endoscopic bilateral metal stent placement for advanced hilar cholangiocarcinoma: a pilot study of a newly designed Y stent. *Gastrointestinal Endoscopy*.

[14] Hwang J. C., Kim J. H., Lim S. G., Kim S. S., Yoo B. M., Cho S. W. (2011). Y-shaped endoscopic bilateral metal stent placement for malignant hilar biliary obstruction: prospective long-term study. *Scandinavian Journal of Gastroenterology*.

[15] Naitoh I., Nakazawa T., Ban T. (2015). 8-mm versus 10-mm diameter self-expandable metallic stent in bilateral endoscopic stent-in-stent deployment for malignant hilar biliary obstruction. *Journal of Hepato-Biliary-Pancreatic Sciences*.

[16] Silverman W., Slivka A. (1996). New technique for bilateral metal mesh stent insertion to treat hilar cholangiocarcinoma. *Gastrointestinal Endoscopy*.

[17] Chahal P., Baron T. H. (2010). Expandable metal stents for endoscopic bilateral stent-within-stent placement for malignant hilar biliary obstruction. *Gastrointestinal Endoscopy*.

[18] Park D. H., Lee S. S., Moon J. H. (2009). Newly designed stent for endoscopic bilateral stent-in-stent placement of metallic stents in patients with malignant hilar biliary strictures: multicenter prospective feasibility study (with videos). *Gastrointestinal Endoscopy*.

[19] Kawakubo K., Kawakami H., Toyokawa Y. (2015). Risk factors for technical failure of endoscopic double self-expandable metallic stent placement by partial stent-in-stent method. *Journal of Hepato-Biliary-Pancreatic Sciences*.

[20] Sofuni A., Maguchi H., Mukai T. (2011). Endoscopic pancreatic duct stents reduce the incidence of post–endoscopic retrograde cholangiopancreatography pancreatitis in high-risk patients. *Clinical Gastroenterology and Hepatology*.

[21] Kim D. U., Kang D. H., Kim G. H. (2013). Bilateral biliary drainage for malignant hilar obstruction using the ‘stent-in-stent’ method with a Y-stent: efficacy and complications. *European Journal of Gastroenterology & Hepatology*.

